# Evaluation of Silent Myocardial Ischemia with Single-Photon Emission Computed Tomography/Computed Tomography in Asymptomatic Subjects with Diabetes and Pre-Diabetes

**DOI:** 10.4274/mirt.24633

**Published:** 2016-06-06

**Authors:** Elif Özdemir, Şefika Burçak Polat, Nilüfer Yıldırım, Şeyda Türkölmez, Reyhan Ersoy, Tahir Durmaz, Telat Keleş, Engin Bozkurt, Bekir Çakır

**Affiliations:** 1 Atatürk Training and Research Hospital, Clinic of Nuclear Medicine, Ankara, Turkey; 2 Atatürk Training and Research Hospital, Clinic of Endocrinology and Metabolism, Ankara, Turkey; 3 Yıldırım Beyazıt University Faculty of Medicine, Department of Nuclear Medicine, Ankara, Turkey; 4 Yıldırım Beyazıt University Faculty of Medicine, Department of Endocrinology and Metabolism, Ankara, Turkey; 5 Yıldırım Beyazıt University Faculty of Medicine, Department of Cardiology, Ankara, Turkey

**Keywords:** type 2 diabetes mellitus, pre-diabetes, silent myocardial ischemia, single-photon emission computed tomography/computed tomography

## Abstract

**Objective:**

The aim of this study was to disclose the prevalence of myocardial ischemia, as detected by adenosine stress myocardial perfusion imaging (MPI) with hybrid single-photon emission computed tomography/computed tomography (SPECT/CT), in asymptomatic diabetic and pre-diabetic patients and to find out whether ischemia predicted the occurrence of adverse cardiac/cerebrovascular events (ACCE) at follow-up.

**Methods:**

Forty-three diabetic and thirty-five pre-diabetic asymptomatic patients without any history of coronary artery disease, underwent MPI and were followed-up for a 12.8±2.2 (8-19) months for the occurrence of ACCE. Baseline variables that would predict the presence of ischemia and the value of ischemia on MPI for predicting the occurrence of ACCE at follow-up were evaluated by logistic regression analysis.

**Results:**

Ischemia was detected in ten (23.3%) of the diabetic and in four (11.4%) of the pre-diabetic patients. The presence of diabetes was the only independent predictor of myocardial ischemia [odds ratio (OR): 12.31, 95% confidence interval (CI): 1.83-82.66; p<0.01]. During 12.8±2.2 (8-19) months of follow-up, ACCE was observed in five out of 78 (6.4%) patients. Patients with ischemia were significantly more likely to have ACCE during follow-up as compared to those with normal MPI scans (event rates: 21.4% vs. 3.1%, OR: 8.455 95% CI: 1.264-56.562, p=0.038).

**Conclusion:**

Myocardial ischemia as detected by adenosine stress SPECT/CT in a population of asymptomatic patients with diabetes mellitus or pre-diabetes appeared to predict the occurrence of ACCE at follow-up.

## Abstract (tr)

**Amaç:**

Bu çalışmanın amacı asemptomatik diyabetik ve pre-diyabetik hastalarda stres miyokardiyal perfüzyon tek foton emisyon bilgisayarlı tomografi/bilgisayarlı tomografi miyokard perfüzyon sintigrafisi (MPS) ile miyokardiyal iskemi prevalansını belirlemek ve iskemi bulgularının takipte istenmeyen kardiyak ve serebrovasküler olayları öngörüp görmediğini saptamaktır.

**Yöntem:**

Daha önce bilinen koroner arter hastalığı olmayan 43 diyabetik ve 35 pre-diyabetik asemptomatik hastaya MPS yapılarak, hastalar istenmeyen kardiyak/serebrovasküler olaylar açısından ortalama 12,8±2,2 (8-19) ay takip edildi. İskemiyi öngören bazal değişkenler ve MPS’nin takipte istenmeyen kardiyovasküler olayları öngörmedeki değeri lojistik regresyon modeli ile değerlendirildi.

**Bulgular:**

Diyabetik hastaların onunda (%23,3) ve pre-diyabetik hastaların dördünde (%11,4) MPS ile iskemi saptandı. Miyokardiyal iskemi açısından tek bağımsız risk faktörü diabetes mellitus varlığı idi (odds ratio (OR): 12,31, %95 güven aralığı (GA): 1,83-82,66; p<0,01). Ortalama 12,8±2,2 (8-19) aylık takip süresi boyunca yetmiş sekiz hastanın beşinde (%6,4) istenmeyen kardiyovasküler olay gelişti. Sintigrafide iskemi bulgusu olanların takibinde kardiyovasküler olay gelişme riski, normal MPS bulguları olanlara göre belirgin olarak yüksekti (olay oranı: %21,4 ile %3,1, OR: 8,455 %95 GA: 1,264-56,562, p=0,038).

**Sonuç:**

Adenozin stres SPECT/CT ile saptanan iskeminin asemptomatik diyabetik ve pre-diyabetik hastalarda takipte gelişecek olan kardiyovasküler olayları öngördüğü söylenebilir.

## INTRODUCTION

Type 2 diabetes mellitus (DM) is associated with an increased risk of coronary artery disease (CAD) and has long been considered a CAD equivalent (1,2). CAD is usually silent in diabetics, and the reported prevalence of silent myocardial ischemia ranges between 6-57% (3,4). This wide range is probably due to differences in patient population and in sensitivity rates of various imaging modalities used in these studies to detect ischemia. Intermediate states of abnormal glucose regulation that exist between normal glucose homeostasis and diabetes are defined as pre-diabetes and include impaired glucose tolerance (IGT), impaired fasting glucose (IFG) or both. Patients with IFG or IGT have a relatively high risk of developing overt diabetes in the future, and CAD risk has been reported to increase in pre-diabetics before glucose levels reach diabetic thresholds (5). Cardio-metabolic derangements occur long before the diagnosis of diabetes and contribute to the 2-fold increased risk of cardiovascular disease seen in IGT and the 4-fold increased CAD risk seen in diabetes (1). According to the American College of Cardiology (ACC) practice guidelines, stress myocardial perfusion imaging (MPI) may be considered for advanced cardiovascular risk assessment in asymptomatic adults with diabetes (class IIb, LoE=C) (6). A similar level of recommendation (class IIb) has been put forward by the European Society of Cardiology for screening selected high-risk patients with DM for the presence of silent myocardial ischemia (7). Quantitative MPI with single-photon emission tomography (SPECT) is a powerful diagnostic modality being used for risk stratification and determination of prognosis in CAD. Recently developed hybrid SPECT/computed tomography (CT) systems were reported to reduce false positive results of myocardial perfusion scintigraphy by eliminating soft tissue attenuation problems and improve the accuracy of MPI (8). In this prospective study, we aimed to disclose the prevalence of silent ischemia, as detected by adenosine stress MPI with hybrid SPECT/CT, in asymptomatic diabetic and pre-diabetic patients and to find out whether ischemia on MPI predicts the occurrence of adverse cardiac/cerebrovascular events (ACCE) at follow-up. We also tried to evaluate the factors that could predict the occurrence of ischemia in this patient subset.

## MATERIALS AND METHODS

We enrolled 35 pre-diabetic and forty-three diabetic patients (mean age=55.6±8.5 years, 35 females) without any history of CAD. The diagnosis of DM and pre-diabetes were made according to American Diabetes Association (ADA) criteria (9). Diabetes diagnosis was based on three fasting blood glucose levels ≥126 mg/dL. Subjects who had fasting plasma glucose levels between 100-125 mg/dL were subjected to 75 gr oral glucose tolerance test (OGTT) where, after an overnight fasting, subjects were given a load of 75 g glucose in 300 mL water. Blood samples for glucose measurements were drawn before the loading and at 120 minutes thereafter. Categories of glucose tolerance were defined according to 2013 ADA criteria (9). IFG was defined as fasting plasma glucose between 100-125 mg/dL and 120 min plasma glucose level <140 mg/dL, whereas IGT was defined as 120 min plasma glucose between 140-199 mg/dL during OGTT. The 120 min glucose level ≥200 mg/dL on 75 gr OGTT were designated as DM. Patients who had either IFG or IGT or both were classified as pre-diabetes. The study was approved by the Hospital Ethical Committee, and informed consent was obtained from all participants before the study procedures.

Exclusion criteria were defined as follows;

1- History of CAD [previous myocardial infarction (MI), percutaneous coronary intervention (PCI) or coronary by-pass graft surgery (CABG)],

2- Electrocardiogram (ECG) findings suggestive of Q wave-MI, ischemic ST-segment or T-wave changes, or complete left bundle branch block,

3- Typical angina pectoris,

4- Absolute contraindication for adenosine stress testing,

5- Segmental wall motion abnormality (WMA) or left ventricular hypertrophy on transthoracic echocardiography,

6- Comorbid conditions that might influence plasma glucose levels such as Cushing syndrome, acromegaly, pheochromocytoma,

7- Use of drugs that may affect plasma glucose levels such as corticosteroids, neuroleptics, antiviral agents, beta agonists, interferon, diazoxide.

After a detailed medical history and a thorough physical examination, a 12-lead ECG and a trans-thoracic echocardiogram were recorded for each patient. Age at onset of diabetes, duration of diabetes and recently used medications were recorded. Major cardiovascular risk factors including smoking, dyslipidemia, hypertension, and family history of CAD were also recorded. Hypertension was defined as systolic blood pressure ≥140 mmHg, diastolic blood pressure ≥90 mmHg, or any treatment with an antihypertensive drug. Microalbuminuria was evaluated on 24-hour urine samples in all patients. The fundoscopic examination was applied to patients with DM.

### Laboratory testing:

Venous blood samples were drawn in the fasting state using vacutainer tubes. Plasma glucose, HbA1c, triglycerides, total and high-density lipoproteins (HDL) and cholesterol concentrations were measured by enzymatic assays (Roche Diagnostics GmbH, Mannheim, Germany). Albumin measurement in 24-hour urine samples was made by immunoradioturbimetric method (Aeroset, Abbott), and levels ≥30 mg/day were defined as ‘microalbuminuria’.

### Myocardial perfusion imaging with SPECT/CT:

All patients underwent same-day rest/stress technetium-99m methoxy-isobutyl-isonitrile (99mTc-MIBI) gated-SPECT MPI by adenosine stress with hybrid SPECT/CT system (Infinia, GE Healthcare). Pharmacologic stress with adenosine was chosen as the stress modality of choice to standardize the stress level and eliminate potential confounding that could arise from different levels of maximum achievable stress due to varying levels of exercise capacity in different patients. For adenosine stress test, patients were asked to refrain from consuming caffeinated beverages after midnight prior to testing. In the morning 296-370 MBq 99mTc-MIBI was injected intravenously at rest and imaging was performed 60 min after injection. Three or four hours after resting acquisition, stress protocol was started. One hundred forty µg/kg per minute adenosine (adenosine-LM, Abfenfarma) was infused intravenously over 4-6 min. 99mTc-MIBI (of 888-1110 MBq) was administered 3 minutes after the beginning of adenosine infusion. After the radiotracer injection, adenosine infusion was continued for another 1-2 minutes. ECG was monitored continuously and blood pressure and heart rate were obtained at 1-min intervals. Stress images were acquired 30-45 minutes after radiotracer injection. The SPECT MPI acquisition was performed on a dual-head camera (Infinia, GE Healthcare) with a low-energy, high-resolution collimator; a 20% symmetric window at 140 keV; a 64x64 matrix; and an elliptic orbit with step-and-shoot acquisition at 3° intervals over a 180° arc (45° right anterior oblique to 45° left posterior oblique) with 30 steps (60 views). Scan time was set to 20 s per frame for stressed and 25 s per frame for resting conditions. Gating included 16 frames per R-R cycle. For attenuation correction (AC), all patients underwent low-dose CT using a Hawkeye system (Infinia; GE Healthcare). Single-slice non-spiral CT (x-ray tube current, 2.5 mA; voltage, 140 kVp) with a slice thickness of 10 mm and a scan time of more than 5 min for a typical 13-cm field of view was obtained. After reconstruction and transfer to a Xeleris workstation (GE Healthcare), AC maps were generated. SPECT images were reconstructed into short and vertical and horizontal long axes using standard reconstruction-that is, filtered back projection-and iterative reconstruction with CT-AC. Visual interpretation of SPECT images was always performed side by side by 2 experienced nuclear medicine specialists. SPECT images were analyzed by using Emory Cardiac Toolbox software package. For semi-quantitative analysis, SPECT images were visually scored using a 20-segment model of the left ventricle and images with AC were evaluated visually and each segment was scored between 0 and 4. Zero was defined as normal perfusion, 1: equivocal, 2: moderate reduction, 3: severe reduction, and 4: lack of perfusion. Summed stress score (SSS) and summed rest scores were obtained by adding the scores of the 20 segments. The summed difference score (SDS) represents the difference between the stress and rest scores. In the semi-quantitative analysis done by using AC images, SSS >1 and SDS >1 were accepted as positive for ischemia (10). Wall motion, wall thickening and left ventricular ejection fraction (LVEF) were evaluated using Quantitative Gated SPECT; (Cedars-Sinai Medical Center) software package.

### Patient follow-up:

The patients were followed up for a mean of 12.8±2.2 (8-19) months for the occurrence of ACCE defined as the occurrence of either typical angina pectoris, MI, myocardial revascularization (PCI or CABG), stroke, cardiovascular or cerebrovascular death, new cardiac arrhythmia or congestive heart failure. Predictors of ischemia in the whole group and in those with DM were evaluated using multiple logistic regression analysis.

### Statistical Analysis

Data analysis was performed by using SPSS for Windows, version 11.5 (SPSS Inc., Chicago, IL, United States). The distribution pattern of metric discrete and continuous variables was evaluated by Kolmogorov-Smirnov test. Metric discrete and continuous variables were shown as the mean ± standard deviation (SD) or median (minimum-maximum), where applicable. The mean value differences between groups were compared by Student’s t-test. Mann-Whitney U test was applied for comparisons of the median values. Nominal data were analyzed by Pearson’s chi-square or Fisher’s exact test, where appropriate. The predictors of ischemia on MPI were evaluated by Multiple Logistic Regression Analyses. Any variable found to be significant on univariate testing (p-value <0.25) was subjected to testing in the multivariate model. Odds ratios and 95% confidence intervals for each independent variable were also calculated. A p value less than 0.05 was considered statistically significant.

## RESULTS

We enrolled 43 diabetics and 35 pre-diabetic subjects. DM had been present for a median of 7 years in the diabetic group. Among the pre-diabetics, 20 had IFG, 6 had IGT and 9 had both. The demographic properties, risk factors and medications that were being used at study entry are shown in Table 1. The two groups (diabetics and pre-diabetics) were similar with regard to age, gender and body mass index. On the other hand, the diabetics were more likely to be hypertensive (50% vs. 11.4%, p=0.001), had higher serum triglyceride levels (170 vs. 130 mg/dl, p=0.002), and were on lipid-lowering therapy more frequently (16.3% vs. 2.9%, p=0.06) as compared to pre-diabetics. Otherwise, the two groups looked similar. Diabetic retinopathy was present in 23.3% of the diabetic population. Microalbuminuria was evident in 14% of the diabetics, and none of the pre-diabetic patients.

### SPECT findings:

**Figure 1:**
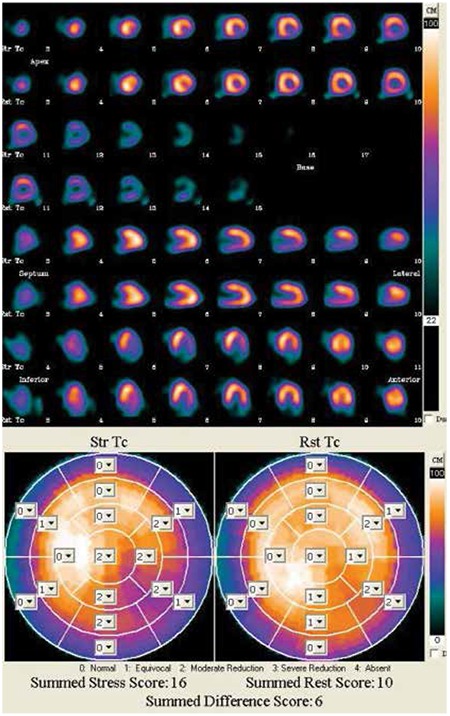
Stress and rest myocardial perfusion single-photon emission computed tomography images of a diabetic patient revealed reversible ischemia in the inferolateral wall

By using 20 segment model, attenuation corrected images, SSS and SDS scores, 4 out of 35 (11.4%) in the pre-diabetics and 10 out of 43 (23.3%) in the diabetic group were found to have ischemia on SPECT/CT MPI (Table 2) (Figure 1). Among the four pre-diabetics with ischemia on MPI, 1 had IFG and 3 had IGT.

Hypo-perfusion involving more than 5% of the left ventricle was detected only in two diabetic patients. Although no patient had WMA on echocardiography at study entry, we detected segmental WMA (all hypokinesia) on post-stress gated images in five diabetic patients on MPI. No patient in the pre-diabetic group had WMA on MPI. The mean LVEF by gated SPECT was 72±7.3% in the pre-diabetic and 70±7.7% in the diabetic group. High SSS and/or SDS scores before AC turned to normal values after AC in 25 patients (31.6%) in the whole group. Table 3 shows the univariate analysis results of all possible predictors of ischemia in the whole group. Those variables found to be significant (p<0.25) on univariate testing were subjected to multivariate analysis, where the presence of DM was found to be the only independent predictor of an abnormal MPI result [odds ratio (OR): 12, 31, 95% confidence interval (CI): 1.83-82.66; p<0.01]. No significant correlation was evident between ischemia on MPI and age, sex, hypertension, microalbuminuria, family history of CAD, smoking, aspirin use, and fasting blood glucose. In the diabetic group, ischemia on MPI was not correlated with hypertension, smoking, family history of CAD, retinopathy, microalbuminuria, treatment modality for DM (oral antidiabetics, insulin, diet), and disease duration. Multivariate analysis revealed a small but a significant correlation between total cholesterol levels and ischemia (OR: 1.035, 95% CI: 1.0005-1.071, p=0.047).

Follow up: During 12.8±2.2 (8-19) months of patient follow-up, we observed ACCE in two (5.7%) pre-diabetic and three (7%) diabetic patients (Table 2). One diabetic patient developed MI and underwent PCI with stenting. This patient had positive scintigraphic findings and his SSS was 8. Four other patients (two diabetics, two pre-diabetic) developed typical angina pectoris and were started on anti-anginal therapy. No patient died because of a cardiovascular or cerebrovascular event during follow-up. Three of the five patients who developed ACCE on follow-up had ischemia on MPI. Patients who had ischemia on MPI were significantly more likely to have ACCE during follow-up as compared to those who had totally normal MPI scans (event rates: 21.4% vs. 3.1%, OR: 8.455 95% CI: 1.264-56.562, p=0.038).

## DISCUSSION

The major findings of this study are;

1- By using adenosine stress MPI with gated SPECT/CT, myocardial ischemia was detected in 23.3% and 11.4% of asymptomatic diabetic and pre-diabetic subjects, respectively,

2- Although the population size and the event numbers are too small to draw any firm conclusions, subjects with ischemia on MPI were significantly more likely to experience ACCE as compared to those with normal scans.

The presence of silent ischemia has been evaluated in diabetic patients utilizing different methods of ischemia detection. In studies using nuclear imaging modalities, perfusion abnormalities were detected in 6-57% of asymptomatic patients with DM (4,11,12,13,14,15,16). In the present study, we evaluated completely asymptomatic patients with diabetes and pre-diabetes without clinically documented CAD. The prevalence of silent ischemia in our study is close to that reported in the Detection of Ischemia in Asymptomatic Diabetics (DIAD) trial (22%), one of the largest trials on asymptomatic ischemia in diabetic patients. Population-based studies report an increased rate of macrovascular complications and mortality in pre-diabetes; and fasting plasma glucose, postprandial plasma glucose and HbA1c are related with increased mortality independent of obesity, hypertension and lipid profile (17,18). Nasr and Sliem (19) reported that pre-diabetics have myocardial defects, which represent a pattern of cardiovascular risk. In our study, the prevalence of hypertension, hypertriglyceridemia and use of statins were more common in the diabetic group as compared to pre-diabetics. Wall motion abnormalities and MPI abnormalities were more frequent in the diabetic group as compared to pre-diabetics but the differences did not reach statistical significance. Our small study population and an even smaller number of pre-diabetics preclude any meaningful discussion on the relative importance of pre-diabetes as opposed to diabetes with regard to the occurrence of asymptomatic ischemia.

Routine screening for silent ischemia in asymptomatic diabetic patients has been a matter of debate for a long time. Guidelines recommend routine screening only for high-risk patients. Janand-Delenne et al. (15) found a positive correlation between silent myocardial ischemia and DM duration, renal status and other classical CAD risk factors except family history. They recommend screening for male patients in whom the duration of DM is 10 years or even less when more than one cardiovascular risk factor is present. In a recent study by Giovacchini et al., (20) microalbuminuria was the only predictor of silent ischemia in diabetes. Gokcel et al. (12) reported a positive correlation between silent ischemia and retinopathy, male sex, hypertension and low HDL-cholesterol levels in patients with DM. In our patient population, which included both diabetics and pre-diabetics, the presence of DM was the only independent predictor of the occurrence of silent ischemia. In the diabetic group, only total cholesterol, neither microalbuminuria nor retinopathy, were found to be significant predictors of ischemia. Our small study sample may have obscured any such possible association, nevertheless it should be kept in mind that diabetic complications do not have to follow a particular sequence. Our findings seem to be in line with those of Peix et al., (21) who showed that the presence of DM was the only predictor of abnormal myocardial perfusion and that total cholesterol/HDL ratio greater than 4 was correlated with perfusion abnormalities. In the DIAD study, where 522 diabetic patients were evaluated with adenosine MPI, an abnormal stress test result was not significantly associated with demographic characteristics, traditional cardiac risk factors, or laboratory variables (4). Anand et al. (22) also screened 510 asymptomatic diabetic patients and did not show any correlation with conventional risk factors and myocardial perfusion abnormalities. In that study, Coronary Artery Calcium (CAC) score was the only predictor of myocardial perfusion abnormality. In light of these findings, they recommended a strategy of initial CAC imaging followed by selective myocardial perfusion scintigraphy (MPS), which has the advantage of combining the high sensitivity rate of CAC imaging with the specificity of MPS for predicting angiographic stenosis.

MPI is a well-established method for the evaluation of CAD and has a high diagnostic accuracy (23,24,25). In our study, we used SPECT/CT for MPI and AC was made with low dose CT and left ventricular function was evaluated in each patient with gated SPECT. The American Society of Nuclear Cardiology and the Society of Nuclear Medicine concluded in their joint position statement that incorporation of AC in addition to ECG gating with MPI would improve image quality, interpretive certainty, and diagnostic accuracy (26). It was shown previously in several studies that AC increased the diagnostic accuracy and contributed to better risk stratification, possibly via decreasing the rate of false positive results (10,27,28,29). In the semi-quantitative analysis, no cut-off values of SSS, SDS and SRS have been established for CT-AC. In a limited number of studies, the cardiac event rate was significantly higher in patients with CT-based AC- SSS between 1-3 compared to the ones with non-AC- SSS between 1-3 (10,29). In our study, we considered SSS or SDS greater than 1 as positive. AC made a great contribution to our study since twenty-five patients (31.6%) with SSS >4 or /and SDS >2 scores in non-attenuation corrected images turned out to have a score of ‘0’ when AC was performed. We believe that the reduction of false positive results on MPI by using AC may prevent unnecessary diagnostic and therapeutic interventions in this patient subset. MPI has been shown to predict the occurrence of adverse cardiac events in asymptomatic diabetic patients (11,12). In a recent study, Kakaletsis et al. (30) used stress MPI in asymptomatic diabetic patients to predict the occurrence of cardiac events at follow-up. They did not use CT for AC and reported that ischemia on MPI was associated with an OR of 3.8 for cardiac event prediction. The OR increased to 7.7 when only extensive ischemia on MPI was taken into account. We observed a significantly higher rate of ACCE (OR: 8.455 95% CI: 1.264-56.562, p=0.038) at follow-up in diabetic and pre-diabetic patients with ischemia on MPI as opposed to those without. The OR that we have found in our study seems to be higher than those reported in the literature. The high rate of cardiac adverse events at follow-up in our study seems to be contradictory to the rate reported in the landmark DIAD trial (6.4% over a mean of 12 months versus 2.9% over 4.8 years, respectively) (4). This discrepancy deserves an explanation and the most likely sources are the small sample size and the simplistic clinical definition of cardiac events in our study. Four out of five cardiac events on follow-up were angina necessitating the institution of anti-anginal therapy. Still, hard end-points like MI appeared to have similar frequencies (1.28% versus 1.3% respectively) in the 2 studies. We believe that when performed by highly accurate methods like the one used in our trial (MPI with gated SPECT/CT), ischemia detection in this patient population may be useful in accurate prediction of the likelihood of ACCE on follow-up, and thus, guide patient selection for more advanced evaluation with respect to CAD. Adenosine SPECT/CT as an imaging modality is a promising tool to detect silent ischemia in diabetic and non-diabetic patients. Whether such a strategy of screening for ischemia in this population will yield clinical improvement still remains to be proven by further studies.

There are some limitations in our study;

1- No control group was included due to ethical reasons,

2- The small population size precluded any meaningful discussion of IGT and IFG groups, even diabetics and pre-diabetics separately,

3- The follow-up interval for cardiac events was rather short.

## CONCLUSION

In conclusion, although the population size and the event numbers are too small to draw any firm conclusions, subjects with ischemia on MPI were significantly more likely to experience ACCE as compared to those with normal scans.

## Ethics

Ethics Committee Approval: The study was approved by the Ankara Atatürk Training and Research Hospital Local Ethics Committee, Informed Consent: Consent form was filled out by all participants.

Peer-review: Externally peer-reviewed.

Financial Disclosure: The authors declared that this study has received no financial support.

## Figures and Tables

**Table 1:**
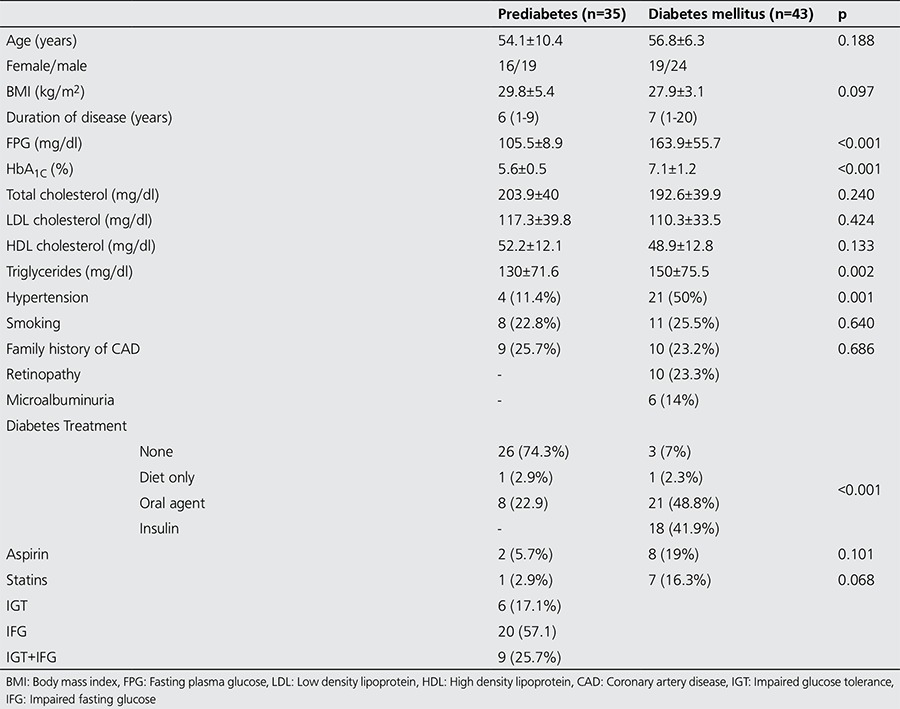
Characteristics of the study population (n=78)

**Table 2:**
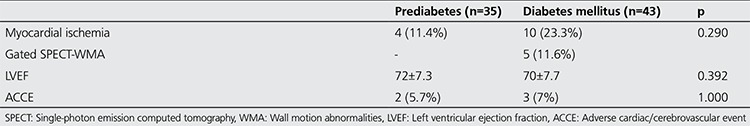
Myocardial perfusion imaging findings and adverse cardiac/cerebrovascular event rates

**Table 3:**
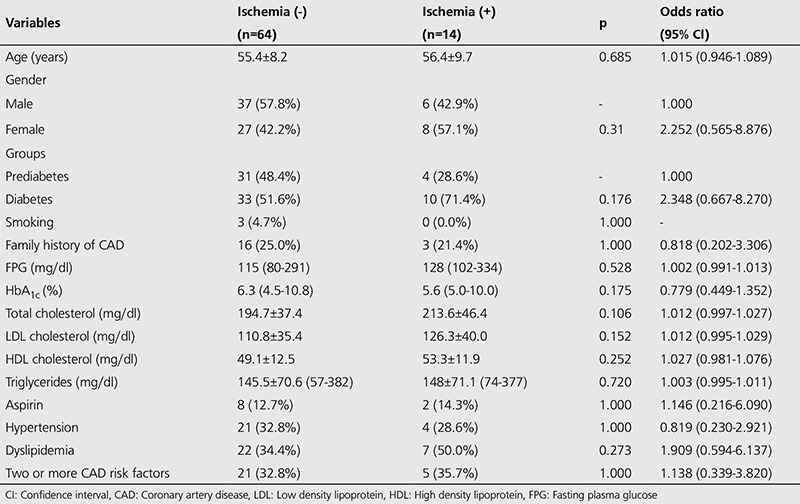
Univariate analysis of the variables predictive of ischemia on myocardial perfusion imaging
